# Seroprevalence of avian hepatitis E virus and avian leucosis virus subgroup J in chicken flocks with hepatitis syndrome, China

**DOI:** 10.1186/s12917-016-0892-4

**Published:** 2016-11-22

**Authors:** Yani Sun, Taofeng Du, Baoyuan Liu, Shahid Faraz Syed, Yiyang Chen, Huixia Li, Xinjie Wang, Gaiping Zhang, En-Min Zhou, Qin Zhao

**Affiliations:** 1 Department of Preventive Veterinary Medicine, College of Veterinary Medicine, Northwest A&F University, Yangling, Shaanxi 712100 China; 2 Scientific Observing and Experimental Station of Veterinary Pharmacology and Veterinary Biotechnology, Ministry of Agriculture, Yangling, Shaanxi 712100 China; 3 College of Animal Science and Veterinary Medicine, Henan Agricultural University, Zhengzhou, Henan 450002 China

**Keywords:** Avian hepatitis E virus, Avian leucosis virus subgroup J, Seroprevalence, Antibodies

## Abstract

**Background:**

From 2014 to 2015 in China, many broiler breeder and layer hen flocks exhibited a decrease in egg production and some chickens developed hepatitis syndrome including hepatomegaly, hepatic necrosis and hemorrhage. Avian hepatitis E virus (HEV) and avian leucosis virus subgroup J (ALV-J) both cause decreasing in egg production, hepatomegaly and hepatic hemorrhage in broiler breeder and layer hens. In the study, the seroprevalence of avian HEV and ALV-J in these flocks emerging the disease from Shandong and Shaanxi provinces were investigated.

**Results:**

A total of 1995 serum samples were collected from 14 flocks with hepatitis syndrome in Shandong and Shaanxi provinces, China. Antibodies against avian HEV and ALV-J in these serum samples were detected using iELISAs. The seroprevalence of anti-avian HEV antibodies (35.09%) was significantly higher than that of anti-ALV-J antibodies (2.16%) (*p* = 0.00). Moreover, the 43 serum samples positive for anti-ALV-J antibodies were all also positive for anti-avian HEV antibodies. In a comparison of both provinces, Shandong chickens exhibited a significantly higher seroprevalence of anti-avian HEV antibodies (42.16%) than Shaanxi chickens (26%) (*p* = 0.00). In addition, the detection of avian HEV RNA and ALV-J cDNA in the liver samples from the flocks of two provinces also showed the same results of the seroprevalence.

**Conclusions:**

In the present study, the results showed that avian HEV infection is widely prevalent and ALV-J infection is endemic in the flocks with hepatitis syndrome from Shandong and Shaanxi provinces of China. These results suggested that avian HEV infection may be the major cause of increased egg drop and hepatitis syndrome observed during the last 2 years in China. These results should be useful to guide development of prevention and control measures to control the diseases within chicken flocks in China.

**Electronic supplementary material:**

The online version of this article (doi:10.1186/s12917-016-0892-4) contains supplementary material, which is available to authorized users.

## Background

From 2014 to 2015, many broiler breeder and layer hen flocks exhibited a decreasing in egg production in some areas of China. Hepatitis syndrome including hepatomegaly, hepatic necrosis and hemorrhage, and retroperitoneal hemorrhage were often occurred in the necropsied chickens from these flocks. All flocks had been vaccinated with Newcastle Disease virus, infectious bronchitis virus, and avian influenza vaccines according to standard industry procedures. Based on a survey of the incidence, clinical symptoms, pathological lesion, and vaccination histories, it can be diagnosed as avian HEV or ALV-J infection.

Avian Hepatitis E virus (HEV) was first characterized in chickens with big liver and spleen disease, also known as hepatitis-splenomegaly (HS) syndrome. The disease mainly causes an increase in mortality, a decrease in egg production and hepatitis syndrome in broiler breeders and laying hens aged from 30 to 72 weeks [1]. In addition, avian HEV has also been shown to cause subclinical infections in chickens [2, 3]. When accompanied by a change in environment and feed or co-infection with other pathogens, subclinically infected flocks may develop big liver and spleen disease and manifest a decrease in egg production. Based on serological evidence, it appears that avian HEV is endemic in the world, even among healthy chicken flocks. For example, in the United States, seropositivity was observed in approximately 71% of flocks (29% of chickens) [4], in Spain in 90% of flocks [2], in Brazil in 20% of chickens [5], and in Korea in 57% of flocks (28% of chickens) [6]. Avian HEV was first characterized in China in 2010 in a report [7].

Avian leucosis virus subgroup J (ALV-J) was first isolated as early as 1988 from meat-type chickens in Great Britain, and soon after it was reported in numerous other areas of the world, including Japan, USA, Argentina, Israel, Malaysia, and China [8–12]. Affected chickens usually exhibit tumor development, depressed immunity, growth retardation, and increased mortality, especially common in broiler breeder hens. In addition, the virus also causes subclinical infections in broiler breeder and layer hens, where infection becomes evident after onset of decreased egg production and hepatic necrosis and hemorrhage [13–15]. In recent years, many Chinese strains of ALV-J have been isolated from white meat-type, egg-type, and Chinese local chickens and this disease has seriously impacted the growth and development of the poultry industry in China [16, 17].

To determine the relationship of egg drop and hepatitis syndrome with avian HEV and ALV-J infections in China, the seroprevalence of the two viruses in these flocks emerging the diseases from Shandong and Shaanxi provinces was investigated, since no vaccines and drugs are available to prevent and treat avian HEV and ALV-J infection in chickens [18, 19]. Furthermore, investigation of the prevalence of the two viruses would also facilitate prevention and control of these infectious diseases. The results indicate that avian HEV infection is prevalent, while ALV-J infection is endemic in some regions of these two provinces. So, avian HEV may be the main causative agent behind the decrease in egg production and hepatitis syndrome in China during the last 2 years. Ultimately, these results should increase our understanding of the impact of avian HEV and ALV-J infection to the Chinese poultry industry.

## Result

### Seroprevalence of antibodies against avian HEV

**Fig. 1:**
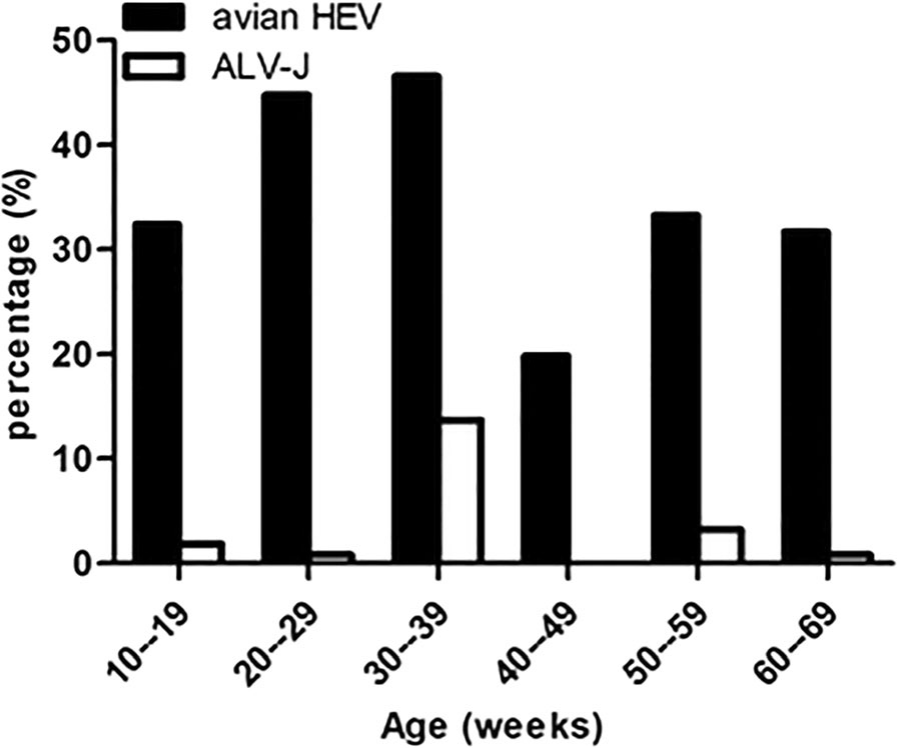
Seroprevalence (%) of avian HEV and ALV-J in chickens within various age groups in Shandong and Shaanxi provinces, China. Anti-avian HEV positivity is presented as *black bars*. Anti-ALV-J positivity is presented as *white bars*

Serum samples were collected from 1995 broiler breeder or layer hens from 14 different chicken flocks located in the Shandong and Shaanxi provinces of China. All flocks exhibited a reduced rate of egg production and hepatitis syndrome. All serum samples were tested for anti-avian HEV antibodies using a previously developed indirect enzyme-linked immunosorbent assay (iELISA) [20] and OD_450nm_ values of these serum samples were determined (Additional file 1). Of 1995 serum samples, 700 (35.09%) were positive for anti-avian HEV antibodies (Table 1). Of these 14 flocks, all were positive for anti-avian HEV antibodies and the proportion of seropositive chickens ranged from 16.67 to 58.44% (Table 1). For comparison of the two provinces, the seroprevalence of Shandong Province (42.16%, 473/1122) was significantly higher than that of Shaanxi Province (26%, 227/873) (*p* = 0.00) (Table 2). When prevalence rates were analyzed at the breed level, the positive rates of avian HEV in broiler breeder hens and layer hens were 34.32% (384/1119) and 36.07% (316/876) respectively. While no significant differences in seroprevalence were observed between broiler breeder hens and layers (*p* = 0.42) (Table 2), significant seroprevalence differences were observed in chickens of different age groups (*p* = 0.00) (Table 2). As shown in Fig. 1, the seroprevalence of different age groups ranged from 19.87 to 46.58%, with highest seroprevalence in the 30 to 39-week-old group (46.58%).

**Table 1:** Seroprevalence of avian HEV and ALV-J infection in chickens of Shandong and Shaanxi provinces, China

District	Flock/County	Type of birds	Age (weeks)	Total	Positive for avian HEV (%)	Positive for ALV-J (%)
Shandong	QDLH/Qingdao	Broiler	16	54	23 (42.59)	1 (1.85)
	YS/Yantai	Broiler	50–58	563	202 (35.88)	24 (4.26)
	XTLH/Heze	Broiler	19	54	12 (22.22)	1 (1.85)
	DL/Weifang	Broiler	>60	70	22 (31.43)	0 (0)
	QLDB/Jinan	Layers	25	243	142 (58.44)	3 (1.23)
	LYLH/Linyi	Broiler	27	138	72 (52.17)	0 (0)
Shaanxi	XP/Xianyang	Layers	50	91	20 (21.98)	0 (0)
	GL/Xian	Layers	50	107	31 (28.97)	1 (0.93)
	TC1/Tongchuan	Layers	69	50	16 (32)	1 (2)
	YL/Yulin	Layers	40–49	192	32 (16.67)	0 (0)
	WG/Xianyang	Layers	35	73	34 (46.58)	10 (13.70)
	PC/Weinan	Layers	21	120	41 (34.17)	1 (0.83)
	AK/Ankang	Broiler	21	120	23 (19.17)	1 (0.83)
	FP/Weinan	Broiler	42	120	30 (25)	0 (0)
Sum					700 (35.09)	43 (2.16)
*p*-value					0.000

**Table 2:** Factors related to anti-avian HEV and ALV-J antibodies positivity among chickens (*n* = 1995)

Characters	Total	Positive for avian HEV (%)	*p*- Value	Positive for ALV-J (%)	*p*- Value
District					
Shandong	1122	473 (42.16)	0.00	29 (2.58)	0.13
Shannxi	873	227 (26)		14 (1.60)	
Type of Birds					
Broiler	1119	384 (34.32)	0.42	28 (2.34)	0.23
Layers	876	316 (36.07)		15 (1.71)	
Age group (weeks)					
10–19	108	35 (32.41)	0.00	2 (1.85)	0.00
20–29	621	278 (44.77)		5 (0.81)	
30–39	73	34 (46.58)		10 (13.70)	
40–49	312	62 (19.87)		0 (0)	
50–59	761	253 (33.25)		25 (3.29)	
60–69	120	38 (31.67)		1 (0.83)

### Seroprevalence of antibodies against ALV-J

The anti-ALV-J antibodies in 1995 serum samples were tested using a commercial ELISA kit (IDEXX Lab Inc.) and OD_650nm_ values of these serum samples were determined (Additional file 2). For these serum samples, 43 were positive for anti-ALV-J antibodies, with a seroprevalence of 2.16% (Table 1) and the seroprevalence rate for flocks was 64% (9/14). Within positive flocks, the proportion of seropositive chickens ranged from 0.83 to 13.70% (Table 1). For the two provinces, the seropositive rate in Shandong was 2.58% (29/1,122) and the rate in Shaanxi was 1.60% (14/873), with no statistically significant difference between provinces (*p* = 0.13) (Table 2). For the broiler breeder and layer hens, seropositive rates were 2.34% (28/1,119) and 1.71% (15/876), respectively, with no statistically significant detectable difference (*p* = 0.23) (Table 2). As observed for avian HEV, statistical significance was observed for anti-ALV-J antibodies seroprevalence in different age groups (*p* = 0.00) (Table 2). As shown in Fig. 1, seroprevalence ranged from 0 to 13.70% in the various age groups, with the highest seroprevalence observed in the 30 to 39-week-old-group (13.70%).

When compared to the seroprevalence of anti-ALV-J antibodies in serum samples, the rate for anti-avian HEV antibodies was significantly higher (*p* = 0.00) (Table 1). In addition, all 43 serum samples that were positive for anti-ALV-J antibodies were also positive for the presence of anti-avian HEV antibodies.

### Detection of avian HEV RNA and ALV-J cDNA from liver samples

To obtain the direct evidence for avian HEV and ALV-J infection in these flocks, avian HEV RNA and ALV-J cDNA were also tested in the 323 liver samples from the flocks using RT-nPCR and PCR, respectively. As shown in Table 3, the 99 (30.65%) and 6 (1.86%) liver samples were positive for avian HEV RNA and ALV-J cDNA respectively. In addition, the positive rate of avian HEV RNA in liver samples from Shandong Province (36.45%, 74/203) was significantly higher than that of Shaanxi Province (20.83%, 25/120) (*p* = 0.002) (Table 3).

**Table 3:** Detection of the liver samples from the diseased chickens in Shandong and Shaanxi province by RT-PCR

District	Number	Positive for avian HEV (%)	*p*- Value	Positive for ALV-J (%)	*p*- Value
Shandong	203	74 (36.45)	0.002	4 (1.97)	0.604
Shaanxi	120	25 (20.83)		2 (1.67)	
Sum	323	99 (30.65)		6 (1.86%)	

## Discussion

Both avian HEV and ALV-J infections have been reported in chicken flocks in China. From 2002 to 2010, ALV-J infection became a common infectious disease in China’s poultry industry [21]. Because of recent worldwide eradication efforts, ALV-J infection now rarely occurs in white-feather broilers, but has gradually returned to local chicken farms in China [22]. Avian HEV was first characterized in 2010 and the damage caused by the virus to China’s poultry industry has only been rarely reported [7]. Because both viruses can cause subclinical and persistent infections in chickens and no vaccines or drugs are available for prevention and treatment, epidemiological investigation and elimination of infected chickens are the only methods currently available for prevention and control.

In the present study, seroprevalence tests showed that avian HEV and ALV-J infections have emerged among broiler and layer flocks in Shandong and Shaanxi provinces, explaining increased egg drop and hepatitis syndrome observed during the last 2 years. The positive rate of anti-avian HEV antibodies was markedly higher than that of ALV-J antibodies (*p* = 0.00). Because some flocks were almost free of ALV-J infection in the sampled areas, we could conclude that avian HEV infection was likely the major cause of the diseases observed among the flocks of broiler breeder and layer hens in China over the last 2 years. In addition, based on the detection of avian HEV RNA and ALV-J cDNA in the liver samples, it was also proved the conclusion (Table 3).

The results demonstrate that avian HEV infection is very common in flocks in Shandong and Shaanxi provinces, but also show that ALV-J infection is endemic in some flocks. The possible reasons for these observations are that avian HEV can easily spread among chickens by the fecal-oral transmission route and no measures have yet been taken to prevent and control the disease in China. However, for ALV-J infection, the disease has been controlled to some degree due to successful eradication programs implemented in China and worldwide after 2010. From these results, it is clear that measures must be taken to control avian HEV infection in non-poultry bird species in China. Interestingly, 43 serum samples positive for anti-ALV-J antibodies were also positive for anti-avian HEV antibodies, but based on the seroprevalence results, it is not clear which virus first infected these 43 chickens. We hypothesize that ALV-J infection can lead to immunosuppression in chickens, which then become more prone to secondary viral infections. Furthermore, both ALV-J and avian HEV can cause persistent infections in chickens. Therefore, further studies will be needed to clarify the potential link between these two viruses and disease pathogenesis in co-infected chickens.

In the present study, the results show that the positive rate of avian HEV infection in the chickens of Shandong Province was higher than Shaanxi Province (*p* = 0.00). This significant difference may be related to differences in quantity, quality, and sources of feed, as well as stocking density and breeding environment. Compared to Shaanxi Province, Shandong is an older poultry industrial region with higher feed tonnage, higher stocking densities, and less satisfactory breeding environments. Therefore, additional epidemiological surveys should be conducted to reveal the relationship between these factors and avian HEV transmission. And the decrease of egg production and increasing of mortality of the sampled flocks in Shandong Province (30–45%, 5–8%) were more severe than in Shaanxi Province (10–13%, 1–3%). The incidence of hepatitis syndrome in sampled flocks in Shandong (92%) was higher than that in Shaanxi (67%). So, the infection extent of avian HEV is positively correlated with damage effect of the disease. In addition, seroprevalence results shown here indicate that anti-avian HEV seroprevalence of the chickens studied in this research in 30 to 39-week group was recorded to be the highest level, with that of 20 to 29-week-old chickens ranking the second highest. The results are consistent with those reported by Huang et al., in which the prevalence of anti-avian HEV antibodies in adult chickens (older than 18 weeks) was higher than in younger ones [23].

## Conclusions

Overall, avian HEV infection is prevalent in broiler breeders and layer hens of Shandong and Shaanxi provinces, China, with avian HEV infection rates markedly higher in Shandong than in Shaanxi Province. Avian HEV infection was likely the main cause of egg drop and hepatitis syndrome in broiler breeder and layer hen flocks in China over the last 2 years. The results suggest that measures must be taken to prevent and control avian HEV infection in chickens, as had been done in the past to successfully control and prevent ALV-J infection.

## Methods

### Data and serum samples collection

With the owners consents, a total of 1995 serum samples were collected from 6 chicken flocks in 6 districts (Yantai, Qingdao, Weifang, Linyi, Jinan and Heze) of Shandong Province and 8 flocks in 6 districts (Yulin, Tongchuan, Weinan, Xianyang, Xian and Ankang) of Shaanxi Province. The flocks from Shandong Province showed the egg production decreasing by 30–45% and mortality increasing by 5–8%. And the flocks from Shaanxi Province showed the egg production decreasing by 10–13% and mortality increasing by 1–3%. The incidence of hepatitis syndrome, including hepatomegaly, hepatic necrosis and hemorrhage, were 92% and 67% in the dead chickens in Shandong and Shaanxi provinces. All of the serum samples were collected from 7 broiler breeder flocks and 7 layer flocks and were divided into the following 6 groups based on the age of the chickens: 10 to 19 weeks, 20 to 29 weeks, 30 to 39 weeks, 40 to 49 weeks, 50 to 59 weeks and 60 to 69 weeks. These serum samples were stored at -20 ^o^C until tested. In addition, total of 323 liver samples were collected from these diseased birds in Shandong (*n* = 203) and Shaanxi provinces (*n* = 120) for detection of avian HEV RNA and ALV-J cDNA. These samples were stored at -80 ^o^C until tested.

Shandong Province is located on the east coast of China, has a semi-humid continental climate, and lies in the warm temperate zone with four distinct seasons. The annual average temperature is 11–14 ^o^C and the average rainfall is 710 mm per year. Shandong is the province with the largest poultry industry in China. In 2011, the output of poultry meat and eggs in Shandong totaled 2.55 and 4 million tons, respectively, accounting for nearly 15% of China’s total output. Therefore, poultry farming in Shandong makes up a large portion of overall Chinese poultry production.

Shaanxi Province is located in central China and also has a warm temperate semi-humid climate with a continental monsoon system and four distinct seasons. Its annual average temperature is 12–14 ^o^C and the average rainfall is 576.9 mm per year. Shaanxi generally lags behind Shandong Province in poultry industry output and thus manages smaller chicken populations.

The animal experiments were approved by the Animal Care and Use Committee of Northwest Agricultural & Forestry University (NWSUAF, Permit Number: AE189056) with adherence to NWSUAF guidelines during handling of all experimental animals.

### iELISA for detecting antibodies in serum samples

To determine whether chickens had been exposed to ALV-J, all serum samples were tested for anti-ALV-J antibodies using a commercial ELISA test kit (FlockChek@ Avian Leucosis Virus Antibody Test Kit-Subgroup J, IDEXX, FlockChek@) according to the manufacturer’s instructions. Absorbance values were measured using an automated ELISA plate reader (Bio-Rad, USA) and the results were analyzed and reported as positive or negative based on the cut-off value listed in the manufacturer’s instructions.

Anti-avian HEV IgG antibodies in the serum samples were tested using iELISA developed by Qin et al. [20]. It employs a recombinant truncated avian HEV ORF2 protein containing the C-terminal 268 amino acids as the coating antigen. The recombinant avian HEV ORF2 protein was expressed in *E. coli* and purified using the BugBuster Ni-NTA His•Bind Purification Kit (Jinsite Co., China). The ORF2 gene originates from a Chinese isolate of avian HEV (CaHEV genotype 3, GenBank number: GU954430). All serum samples were tested in duplicate for each of the two iELISAs to test for anti-ALV-J and anti-avian HEV antibodies.

### Detection of viral RNA or cDNA from liver samples

To obtain the direct evidence of avian HEV and ALV-J infection in these flocks, the avian HEV RNA and ALV-J cDNA were tested in the liver samples from these flocks by RT-nPCR and PCR. Total viral RNA and DNA were extracted with EasyPure Viral DNA/RNA kit (Beijing TransGen Biotech, Ltd., China) from 323 liver samples according to the manufacturer's instructions. The partial ORF2 gene (242 bp) of avian HEV RNA was detected from the extracted RNA and DNA by RT-nPCR same as described by Sun et al. [2]. And for the detection of ALV-J, it was expected to give a 545 bp product by the detection of a well conserved region of the gp85 in amplifications by PCR as described by Smith et al. [24].

### Statistical analyses

The seroprevalence values of avian HEV and ALV-J in the chickens were calculated for the total study population. Data collection and analysis were performed using IBM SPSS Statistics 20. Chi-squared tests (*χ*2) for independence or for trend were used to assess the association of categorical variables and anti-avian HEV or anti-ALV-J antibodies status. The results were considered statistically significant when *p* < 0.01.

## Additional files

Additional file 1: OD_450_ values of anti-avian HEV IgG antibodies in the serum samples collected from Shandong and Shaanxi provinces. (The cut-off value is 0.368). (XLS 218 kb)
Additional file 2: OD_650_ values of anti-ALV-J IgG antibodies in the serum samples collected from Shandong and Shaanxi provinces. [S/P<=0.6: Negative; S/P>0.6: Positive. S/P= (Average of sample – Average of negative control)/(Average of positive – Average of negative)]. (XLS 82 kb)

## Abbreviations

ALV-J: Avian leucosis virus subgroup J
ELISA: Enzyme-linked immunosorbent assay
HEV: Hepatitis E virus
ORF2: Open reading frame 2
